# Amplicon Characterization of Fungal Diversity and Functional Prediction of Glycine max L. Rhizosphere Microbiome Under Different Organic Fertilization

**DOI:** 10.1002/mbo3.70437

**Published:** 2026-10-08

**Authors:** Ijeoma Emelda Osuji, Akinlolu Olalekan Akanmu, Olubukola Oluranti Babalola

**Affiliations:** ^1^ Soil‐Plant‐Microbe Interactions Group, Faculty of Natural and Agricultural Sciences North‐West University Mmabatho South Africa; ^2^ Department of Biological Sciences, College of Basic Sciences Lagos State University of Science and Technology Ikorodu Lagos Nigeria; ^3^ Department of Life Sciences Imperial College London Ascot Berkshire UK

**Keywords:** amplicon sequencing, fungal diversity, ITS rRNA, organic fertilization, soil health

## Abstract

The rhizosphere microbiome is extremely important for soil health and plant productivity, with fungi primarily acting as the drivers of soil ecosystem functions. This study explores the fungal diversity and predicted functional potential of the soybean (Glycine max L.) rhizosphere under different organic fertilization treatments, including cattle dung, poultry waste, untreated control, and bulk. Fungal communities were profiled using ITS rRNA gene amplicon sequencing on the Illumina NovaSeq. 6000 platform following DNA extraction from rhizosphere soil. Raw sequences were processed and analyzed using QIIME 2 version 2019.1. Significant differences in fungal richness were observed among treatments. Bulk soil and cattle dung‐amended soils showed the highest richness (Chao1 = 1603.33 and 1456.64, respectively), whereas poultry waste‐amended soil exhibited the lowest richness (Chao1 = 957.93) and the lowest evenness (Pielou's index = 0.560), and the untreated control showed intermediate richness (Chao1 = 1242.11). The dominant phyla include Ascomycota, Basidiomycota, and Zygomycota, with enrichment in organic matter degradation and plant symbiosis. Beta‐diversity analysis showed the greatest compositional divergence between cattle dung and poultry waste (ANOSIM *R* = 0.74), none of the pairwise comparisons reached conventional statistical significance (*p* > 0.05), indicating ecologically relevant but statistically subtle shifts in community structure. LEfSe identified Hypocreales as a significant biomarker of cattle dung and Onygenales of poultry waste, consistent with FUNGuild predictions of enriched dung/wood‐saprotrophic and keratinolytic functions, respectively. FUNGuild predictions showed increased saprotroph and symbiotroph diversity in organically amended soils. Organic fertilizers enrich beneficial fungi, enhancing ecosystem functions in sustainable soybean cultivation.

## Graphical abstract

The rhizosphere microbiome is important for soil health and plant productivity, with fungi primarily acting as drivers of soil ecosystem functions. The profiling of community composition across treatments with whole‐amplicon sequencing of the fungal ITS region was conducted. Organic fertilizers enrich beneficial fungi, enhancing ecosystem functions and sustainable soybean cultivation.

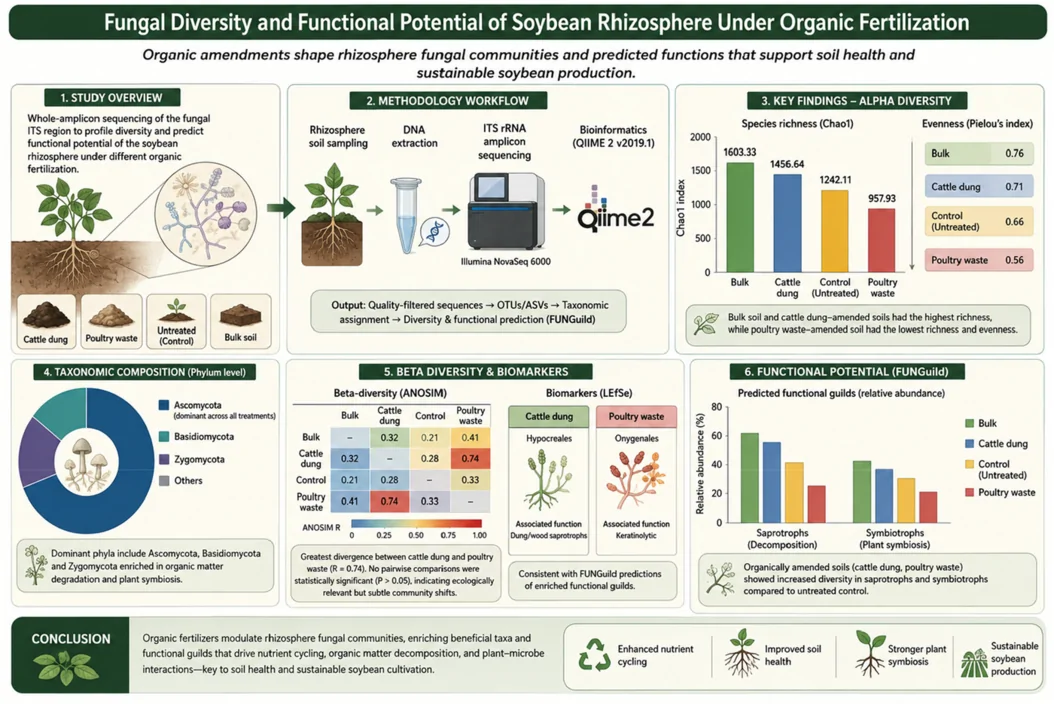

## 1 Introduction

Soybean (Glycine max L.), also known as the Golden bean, is one of the most lucrative, adaptable, and nutritionally important legumes across the globe (Rastogi et al. 2023). It is cultivated over a range of environments and under several management practices (Shea et al. 2022). It is an essential crop that plays a key role in agriculture and global food security (Ajiboye et al. 2022). It is a major source of plant‐based protein and oil, making it a crucial component of human and animal diets (Shea et al. 2022). Soybean production is vast, with the global production reaching over 360 million metric tons in 2021 (Wei et al. 2023).

The soybean rhizosphere, which is a vibrant hub of microbial activity with bacteria, fungi, and other microorganisms, supports the health of the soil and plant life. Thus, the rhizosphere is the narrow zone of most intense bacterial activity around the roots of the Leguminosae (Zhou et al. 2020). It has been reported that the rhizosphere and bulk soil possess different microbial composition, as the rhizosphere harbours elevated numbers of active microorganisms compared to the bulk soil due to increased nutrient availability in the rhizosphere (Bakhshandeh et al. 2020). Due to the possible benefits, such as building food classification via supporting production of two or more crops grown concurrently in the same area, cereal and legume intercropping is vital (Glaze‐Corcoran et al. 2020; Babalola et al. 2025a). Over time, soil fertility and, consequently, soybean production have declined as a result of the continuous use of chemical fertilizers (Fontana et al. 2021). Hence, current agricultural practices must be considerably modified to reduce the use of excessive chemicals and their impact on the environment, while still maintaining high crop yields.

Different studies have shown that soil microbiota around the plant roots significantly contribute to soil fertility levels (Hartmann et al. 2008; Saleem et al. 2018). Plant exudates shape the root microbiome, fostering microbial associations in the rhizosphere. They provide nutrients, create niches, protect against pathogens, produce hormones and aid in plant adaptation to environmental stressors like drought (Enagbonma et al. 2023). The use of chemical‐based products in farm management often affects the ecological niche in the long term or with excessive use, thereby causing a decline in the quality and quantity of soybean yield produced (Majidian et al. 2024; Akanmu et al. 2023). Organic cultivation of soybeans is a sustainable farming practice that involves using organic fertilizers, such as manure, compost and green manure, instead of chemical fertilizers. It promotes soil health, reduces environmental pollution, and enhances the nutritional quality of the crop (Wei et al. 2023). The long‐term application of organic fertilization has been shown to increase soil organic matter, improve soil structure and promote the growth of beneficial microbes, such as rhizobia, which can fix atmospheric nitrogen and make it available to plants (Deng et al. 2012; Wei et al. 2023).

Soybean cultivation faces challenges from organisms like soybean cyst nematode (SCN) (Heterodera glycinea), Phytophthora Root and Stem Rot (Phytophthora sojae), Frogeye Leaf Spot (Cercospora sojina), sudden death syndrome (SDS) (Fusarium virguliforme), Brown Stem Rot (Phialophora gregata), Charcoal Rot (Macrophomina phaseolina), Downy Mildew (Peronospora manshurica), Soybean Rust (Phakopsora pachyrhizi), Anthracnose (Colletotrichum truncatum and other species), and Stem Canker (Diaporthe phaseolorum var. caulivora) (Wei et al. 2023). Traditional microbial analysis relied on culturing methods, but over 95% of soil microbial species are non‐culturable (Giagnoni et al. 2018). Advancements in next‐generation sequencing, such as ITS amplicon sequencing, are revolutionizing our understanding of soil microbial diversity, interactions, and functions (Mishra et al. 2023). Despite these advances, the diversity and functions of rhizosphere microbiomes, especially in the rhizosphere of soybean grown under organic conditions, remain largely unexplored. Several benefits have been accrued from the use of organic amendments sourced from cattle dung and poultry litter wastes, and this involves the improvement of soil structure by stimulating microbial activity, which in turn enhances soil aggregation, increases water retention, and promotes long‐term fertility (Rastogi et al. 2023; Akanmu et al. 2021). Moreover, discovery and understanding of their roles in the microbe‐plant association hold key importance for further exploration towards sustainable soybean production. Progress in metagenomics research has facilitated the use of ITS amplicon sequencing to analyze environmental DNA, thereby offering a more comprehensive understanding of the diversity of the soil microbiome and its functional roles within the ecosystems (Mishra et al. 2023; Babalola et al. 2025b).

Due to the importance of rhizosphere communities for plant growth and performance, various investigations have been carried out using both culture‐dependent and culture‐independent approaches (Fadiji et al. 2021). Despite these advances, the dynamics of fungal microbes associated with soybean rhizosphere grown under different organic conditions remain largely unexplored. Hence, this research investigates the soybean rhizosphere fungal community structure and its functional diversity under poultry waste and cattle dung manure amendment, untreated control, and bulk soils. To our knowledge, this is among the first studies to combine ITS amplicon profiling with FUNGuild‐based functional prediction to directly compare cattle dung and poultry waste organic amendments in the soybean rhizosphere, thereby providing a novel, amendment‐specific perspective that extends beyond previous comparisons of organic versus inorganic fertilization in this crop.

## 2 Materials and Methods

### 2.1 Experimental Layout

The samples were collected at the cultivated soybean plot at the North‐West University farm in Molelwane, Mafikeng, South Africa (25° 48′ 11.577″S, 25° 35′ 53.7 62″E), which has an average rainfall of 540 mm per year. The field experiment was laid out as a randomized complete block design (RCBD), in which the soil type amended with poultry manure, cattle dung, and unamended soil (soybean rhizosphere soil as a control) and bulk soil (non‐rhizosphere soil) was tested in three replications, each plot being 3 m × 3 m (9 m^2^). Adjacent plots were separated by 0.5 m, and blocks were separated by 1 m to reduce the interference of treatments. A pre‐planting rate of 20 t ha^−1^ (2 kg m^−2^) of organic amendments was used. The soybean seeds (cultivar PAN 1555 R) were planted according to conventional practices.

### 2.2 Soil Sample Collection

Rhizosphere soil samples were collected at the flowering stage (7th week after planting), representing the peak of root‐microbe interaction. These soil samples were collected in three replicates from the rhizosphere soil. With the exception of the bulk soil, all soil samples (cattle dung‐amended, poultry waste‐amended, and untreated control) were collected as rhizosphere soil, defined as soil tightly adhering to the root surface after gentle shaking to remove loosely attached soil. Bulk soil samples were collected separately from open inter‐row areas at least 20 cm away from the nearest soybean root system and had no contact with root surfaces, thereby representing non‐rhizosphere soil used for comparison. The collected samples were transported in cooler boxes containing ice to the laboratory, and soil samples were divided into two parts. The first part was stored at −20°C until soil DNA extractions were completed, and the second part was subjected to physicochemical analysis revealing notable enrichment of nutrients under poultry waste (P and Ca) and cattle dung (P, K, and Mg) treatments (Table S1).

### 2.3 DNA Extraction and Sequencing

DNA was extracted from the soil using a NucleoSpin Soil Mini kit for soil DNA isolation following the manufacturer's instructions. A NanoDrop spectrophotometer (Thermo Fisher Scientific, CA, USA) was used to determine the DNA concentration. ITS RNA gene amplicon sequencing of the DNA samples was carried out at the Novogene Laboratory, Singapore.

### 2.4 Sequencing and Data Processing

The internal transcribed spacer (ITS1 and ITS2) regions of the fungal ribosomal RNA gene were amplified using the universal primer pairs ITS1F (CTTGGTCATTTAGAGGAAGTAA) and ITS2 (GCTGCGTTCTTCATCGATGC). Amplicon metagenomic sequencing was carried out using the NovaSeq. 6000 system (Novogene, Singapore). Quantified libraries were pooled and sequenced on Illumina platforms (Bokulich and Mills 2013), following standard protocol. Data obtained from this analysis were used to determine the structural diversity and functional profiles of fungi in the soybean rhizosphere across the treatments. The raw sequence dataset in FASTQ format was demultiplexed using the Divisive Amplicon Denoising Algorithm 2 (DADA2) plugin within QIIME version 2.1 (Bolyen et al. 2019). A pre‐trained Naïve Bayes classifier was applied for ITS using the Unite_INSDC version 8.0 (2018) as a reference database (Bokulich et al. 2018). FUNGuild (Tanunchai et al. 2023) was employed for the functional annotation of the ITS rRNA genes. Downstream analyses included diversity metrics (alpha and beta diversity), visualization of community composition, and phylogenetic tree construction using the SATé‐Enabled Phylogenetic Placement (SEPP) (Tsang et al. 2024) plugin for accurate placement of Amplicon Sequence Variants (ASVs) within the phylogenetic framework. The pipeline facilitated robust and reproducible insights into microbial community structure and function.

The dataset is made up of raw sequence data available in fastq.gz obtained through Amplicon sequencing of the soybean rhizosphere metagenome. All the obtained datasets in fastq.gz file were deposited at the NCBI‐SRA database with accession number SRP541849.

### 2.5 Statistical Analysis

One‐way analysis of variance (ANOVA) was used to test the differences between treatments with the physicochemical data. Tukey's Honest Significant Difference (HSD) test at a 95% confidence level (*p* < 0.05) was used for mean separation. Data were analyzed using the SAS software (version 9.1). The sequence dataset was processed through QIIME 2, using the default parameters. The richness and diversity indexes (Shannon‐Weaver, Evenness index, and Chao 1) were calculated for each pre‐processed data set with R version 3.4.1. Canoco version 5.0 was used to perform the principal component analysis (PCA) and Principal Coordinates Analysis (PCoA).

Three field replicates per treatment were used, consistent with standard practice for resource‐intensive soil‐amplicon microbiome trials of this scale and comparable to the replication levels reported in similar organic amendment fungal community studies (e.g., Gao et al. 2022; Iqbal et al. 2022). A formal post hoc power analysis was not performed, as the small effective sample size (*n* = 3) limits the reliability of power estimates for multivariate community‐level metrics. However, we acknowledge that this replication level constrains statistical power to detect subtle beta‐diversity and community‐composition differences, which likely contributes to the non‐significant ANOSIM and MRPP outcomes reported despite moderate R‐values.

## 3 Results

### 3.1 Microbial Community Composition

The heatmap at the phylum level in Figure 1 illustrates the relative abundance of dominant fungal phyla across soils treated with cattle dung, poultry waste, control, and bulk soil samples. Cattle dung‐amended soils demonstrated a higher relative abundance of Chytridiomycota, Aphelidiomycota, and Rozellomycota. Poultry waste treatment exhibited a higher abundance of Mortierellomycota and Ascomycota. The control (untreated) and bulk soils were characterized by elevated levels of Olpidiomycota, Zoopagomycota and Kickxellomycota, reflecting a relatively less diverse but more stress‐tolerant fungal community (Figure 1a). At the genus level, the fungal community structure across the treatments shows that cattle dung was abundant with Chaetomium, Neurospora, Achaetomium, and Humicola (Ascomycota); poultry waste with Trichoderma and Penicillium; while untreated control and bulk treatment exhibited dominance of Fusarium, Curvularia, and Stagonosporopsis (Figure 1b).

**Figure 1:**
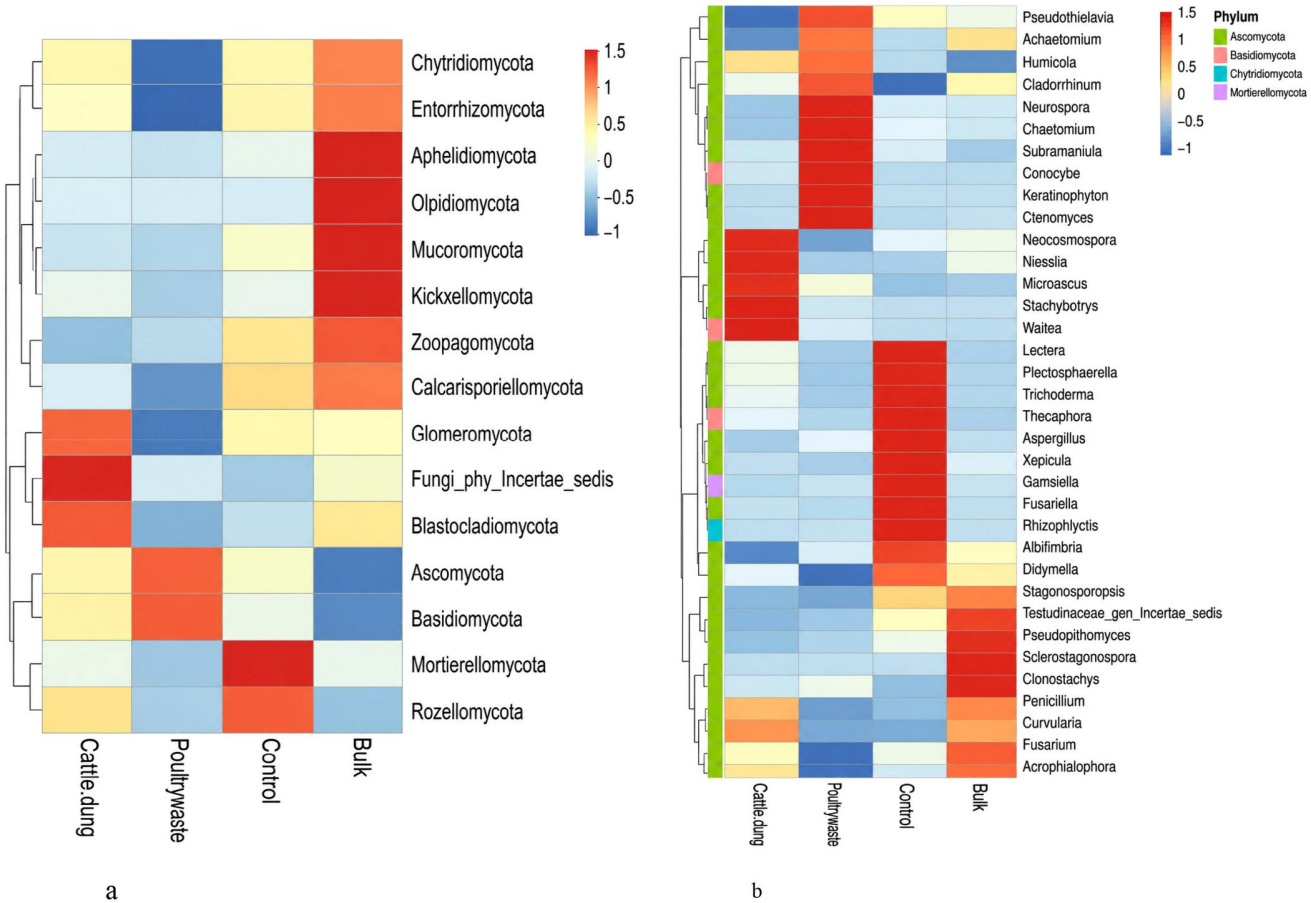
Heatmap showing the relative abundance of fungal phyla across different treatments (cattle dung, poultry waste, control, and bulk soil).

The relative abundance of dominant fungal genera further corroborated these community‐level patterns (Figure 2). Poultry waste soils were characterized by a marked dominance of Keratinophyton, consistent with the keratin‐rich substrate. Cattle dung soils supported a more diverse distribution dominated by Fusarium, Aspergillus, and Subramaniula, while the control and bulk soils exhibited more balanced communities across a broader range of genera at moderate abundance. This indicates that organic fertilization exerts a genus‐specific selective effect on the resident fungal community.

**Figure 2:**
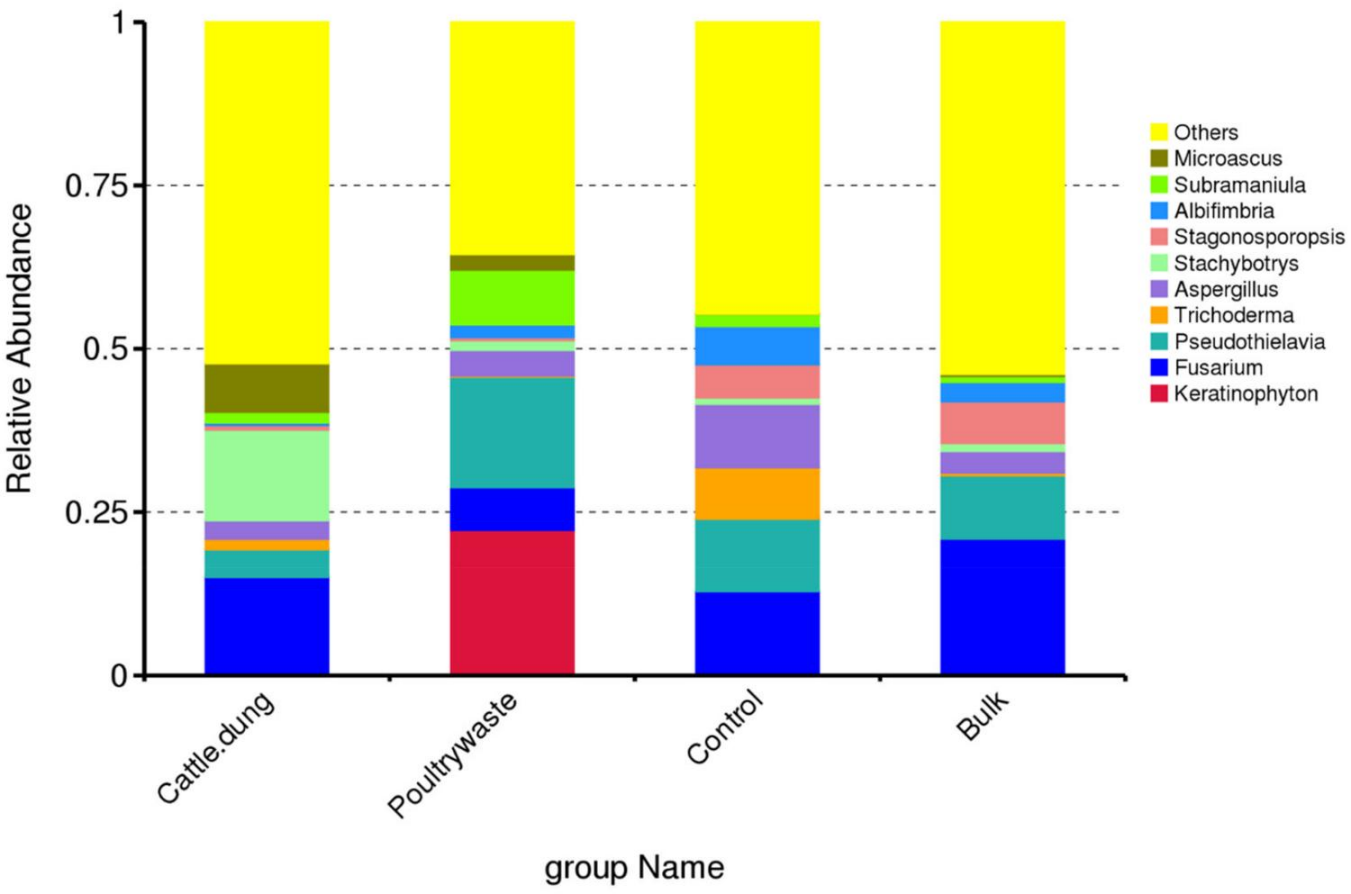
Relative abundance of dominant fungal genera across treatments.

Sequencing yielded high‐quality reads across all treatments, with an average of 165,000–172,000 non‐chimeric reads per sample and Q20 and Q30 values above 99% and 97%, respectively, confirming adequate sequencing depth and quality for downstream diversity analyses; complete read‐processing statistics are provided in Table S2.

Venn diagram analysis of amplicon sequence variants (ASVs) showed that each treatment harboured a distinct fraction of unique taxa: bulk soil (756 ASVs) and cattle dung (655 ASVs) contained the largest numbers of unique ASVs, followed by poultry waste (330 ASVs) and control (476 ASVs), with a core microbiome of 300 ASVs shared across all treatments (Figure 3a). This shared core, together with several pairwise and three‐way overlaps, indicates a stable baseline fungal community alongside treatment‐specific taxa. The corresponding phylogenetic placement of genera (Figure 3b) shows that taxa from Glomeromycota, Chytridiomycota, Mortierellomycota, and Basidiomycota were associated mainly with the organically amended soils, with cattle dung enriched for Ascomycete genera such as Fusarium, Trichoderma, and Humicola, and poultry waste enriched for Basidiomycota genera such as Coprinellus and Conocybe, while control soils remained dominated by Ascomycota (e.g., Cladosporium, Alternaria).

**Figure 3:**
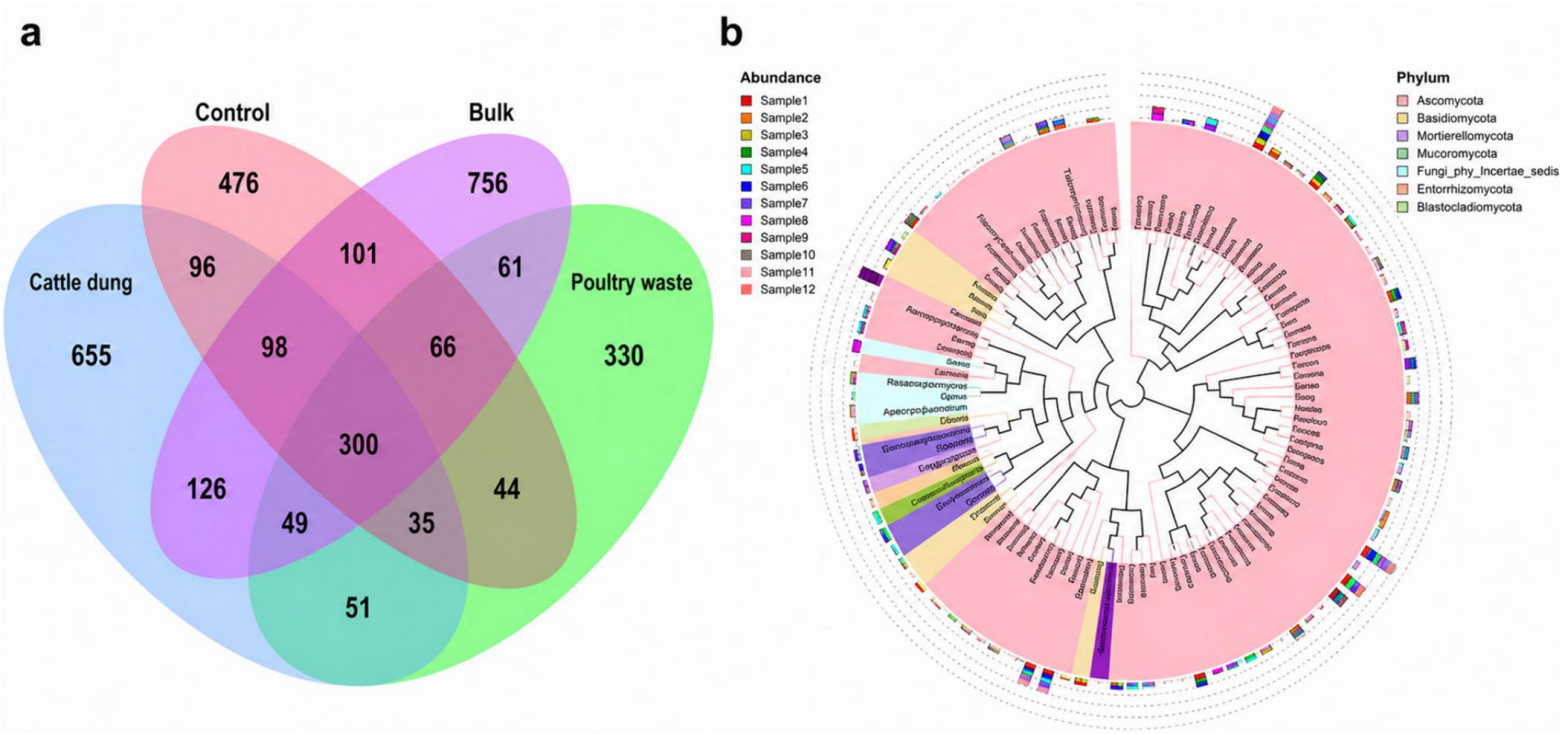
(a) Venn diagram representation of the microbial community and (b) phylogenetic placement of fungal genera in the soybean rhizosphere under various soil treatments.

In Figure S3a, differences in microbial abundance between cattle dung and poultry waste are indicated by differences among the observable genera; poultry waste maintained greater growth of microbes, with genera such as Coccimicrococcus, Nocardiopsis, Rhodococcus, and Cereibacter enriched relative to cattle dung. In Figure S3b, genera Gemmata, Iamia, Sciscionibacter, and Bosephila were highly concentrated in bulk soil, indicating that organic amendment enhances the growth and activity of microbes relative to the undisturbed bulk soil.

The ternary plots uncovered specific distinctions within treatment combinations regarding the distribution of dominant fungal genera (Figure 4). In the comparison of cattle dung, poultry waste, and control (untreated soils) (Figure 4a), Hypocreales and Glomerellales are positioned closer to poultry waste, and Pezizales appear more concentrated towards the control, indicating less influence by organic inputs and representing a natural soil background community. Eurotiales and Microascales are dispersed between cattle dung and control, while Sordariales, although less dominant, tend to spread between cattle dung and poultry waste. When cattle dung, poultry waste, and bulk soil are compared (Figure 4b), Sordariomycetes were highly abundant with a stronger presence in bulk soil compared to cattle dung and poultry waste treatments. Dothideomycetes favour poultry waste, while Eurotiomycetes favour control and bulk, implying less amended soils. Mortierellomycetes and Pezizomycetes are minor contributors that are more common in cattle dung and bulk treatments. Fewer but varied ecological functions of Ustilaginomycetes and Tremellomycetes are found scattered throughout treatments.

**Figure 4:**
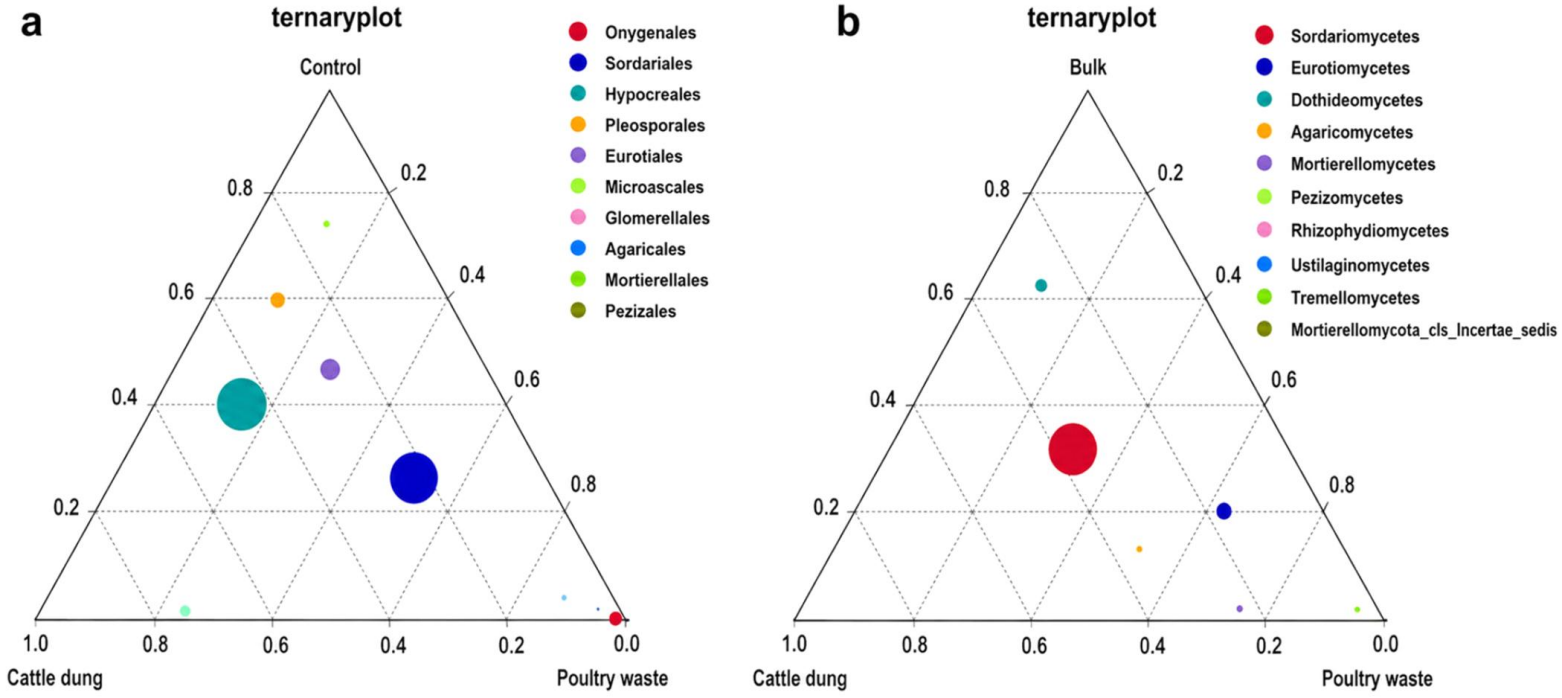
Ternary distribution of dominant fungal phyla across soil treatments: (a) cattle dung, poultry waste and control; (b) cattle dung, poultry waste and bulk soil.

### 3.2 Diversity Metrics

#### 3.2.1 Alpha Diversity of Fungal Communities

The alpha diversity indices in Table 1 revealed notable variations in fungal richness and evenness among treatments. Bulk soil exhibited the highest richness with Chao1 richness (1603.33) and observed features (1557), followed by cattle dung (Chao1: 1456.64), while poultry waste showed the lowest richness (Chao1 = 957.93). Good's coverage remained consistently high (1.000) across all treatments. Diversity indices such as Shannon and Simpson were highest in cattle dung (6.561; 0.975) and bulk soil (6.528; 0.970), while poultry waste treatment exhibited lower Shannon (5.525) and Pielou's evenness (0.560). Bulk soil and cattle dung treatment (0.616; 0.627) revealed higher evenness compared to poultry waste (0.560) and control soils in Pielou's evenness (Figure 5a); Shannon diversity shows greater diversity in cattle dung (6.561) and bulk (6.528) soils (Figure 5b), followed by the Simpson index with high values in cattle dung (0.975) and bulk soils (0.970) (Figure 5c).

**Table 1:** Alpha diversity of fungal communities under different soil treatments.

Sample name	chao1	Dominance	Goods coverage	Observed features	pielou_e	Shannon	Simpson
Cattle dung	1456.642	0.025	1.000	1410	0.627	6.561	0.975
Poultry waste	957.925	0.058	1.000	936	0.560	5.525	0.942
Control	1242.107	0.036	1.000	1216	0.598	6.131	0.964
Bulk	1603.329	0.030	1.000	1557	0.616	6.528	0.970

**Figure 5:**
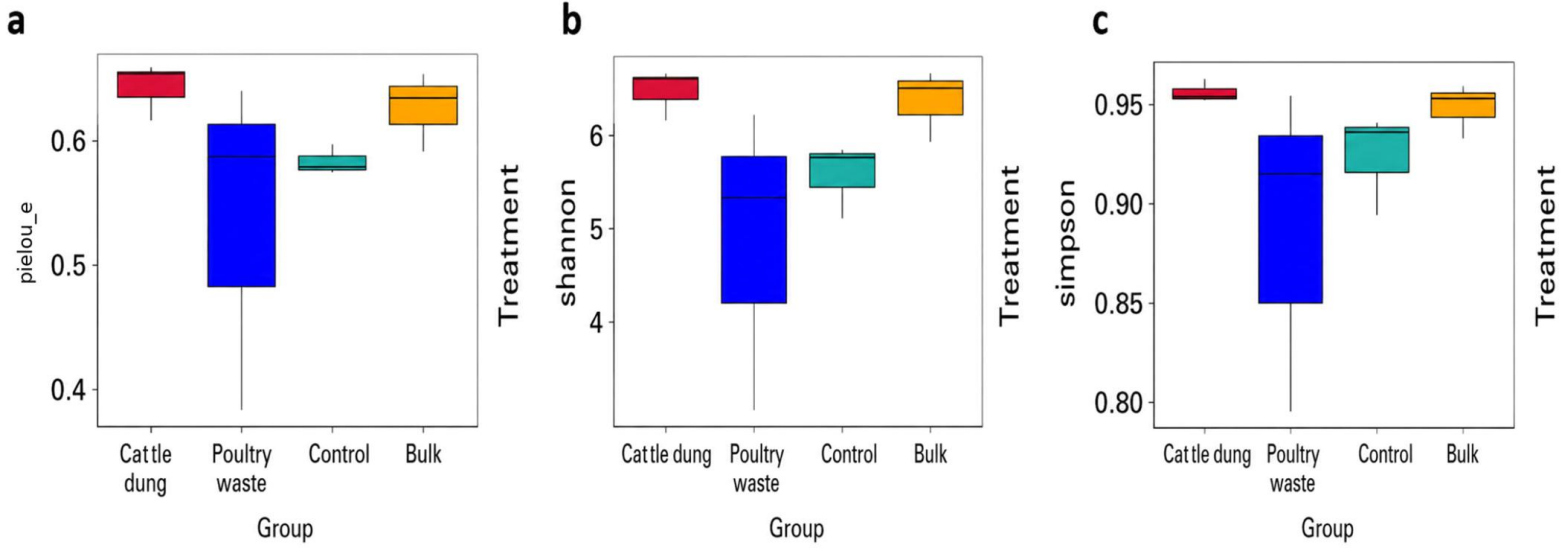
Boxplots of fungal community diversity indices of soybean rhizosphere showing (a) pielou's evenness, (b) shannon diversity, and (c) simpson index. Chao1 richness values are reported in Table 1.

#### 3.2.2 Beta Diversity and Community Dissimilarity

Pairwise beta diversity compares the variations in the decomposition of the fungi community among different treatments (Figure 6). Beta diversity values measured the degree of dissimilarity among various treatments, with a corresponding statistical value that indicates the strength of these differences. The highest dissimilarity was observed between poultry waste and control (*β* = 0.370; stat= 0.596), suggesting strongly reshaped microbial composition compared to control and bulk. Similarly, cattle dung and poultry showed the most divergence (*β* = 0.353; stat= 0.500), reflecting the differential impact of organic amendments on fungal taxa. Moderate dissimilarities occurred between cattle dung and bulk soil (*β* = 0.347; stat 0.509). The dissimilarity between bulk soil and control (*β* = 0.240; stat 0.546) was relatively lower, indicating that these two groups harboured more comparable microbial profiles, likely due to the absence of strong organic inputs.

**Figure 6:**
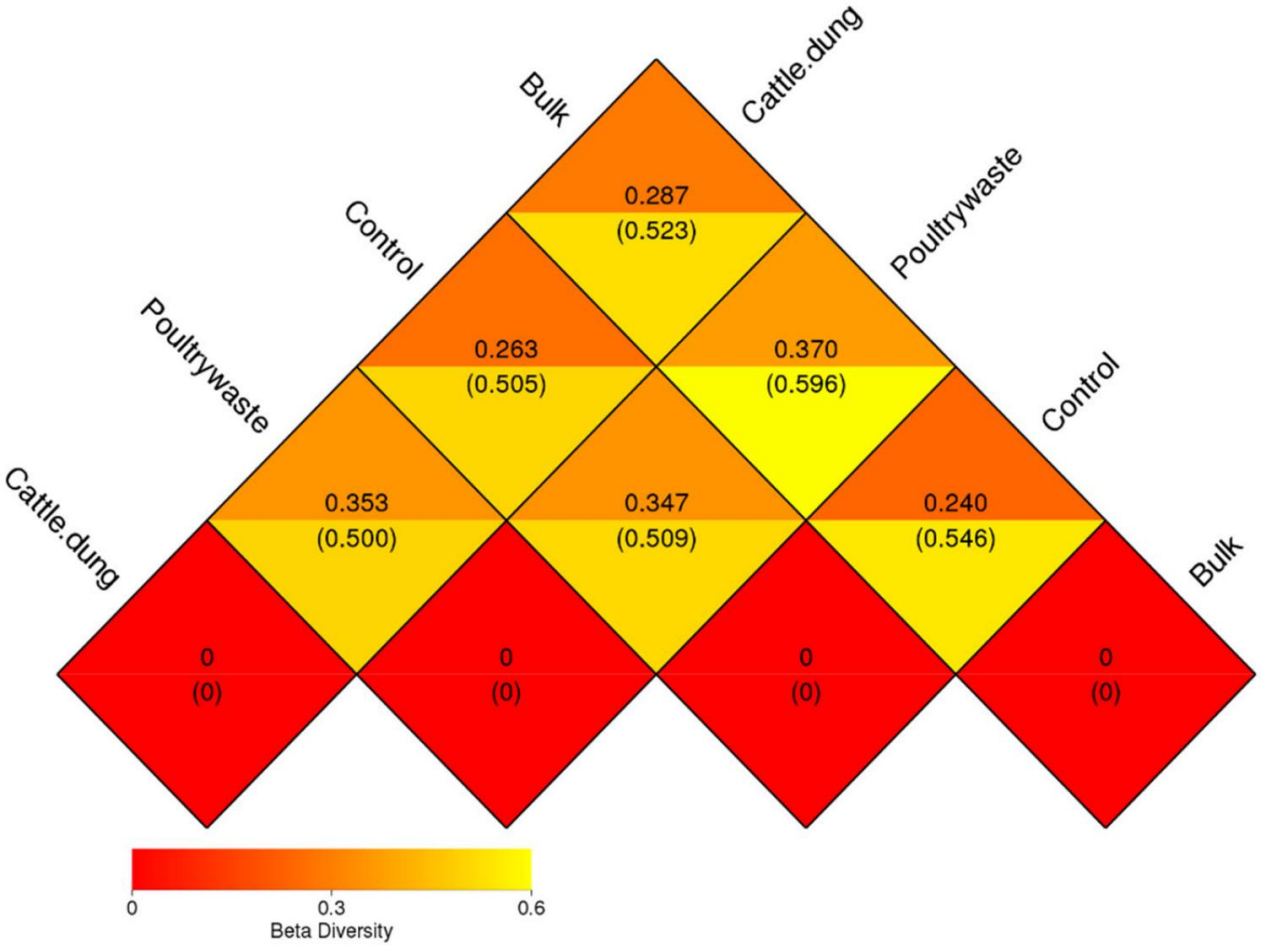
Beta diversity of fungal community of soybean rhizosphere.

#### 3.2.3 Multivariate Analysis of Microbial Community Structure

PCA visualizes differences in fungal community structures across treatments. The first two components, PC1 (13.02%) and PC2 (11.83%), explained 24% of the total variance in the community composition. Clear clustering patterns were observed between treatments. On the negative axis of PC1, samples of cattle dung were specifically clustered from each other, indicating the uniqueness of these microbial assemblages compared with other treatments. Furthermore, poultry waste samples were separated towards the positive side of PC1, which indicates that poultry waste amendments supported a divergent community structure. Samples of bulk and control soil, on the other hand, spread out more on both axes, thereby revealing the heterogeneous community composition (Figure 7).

**Figure 7:**
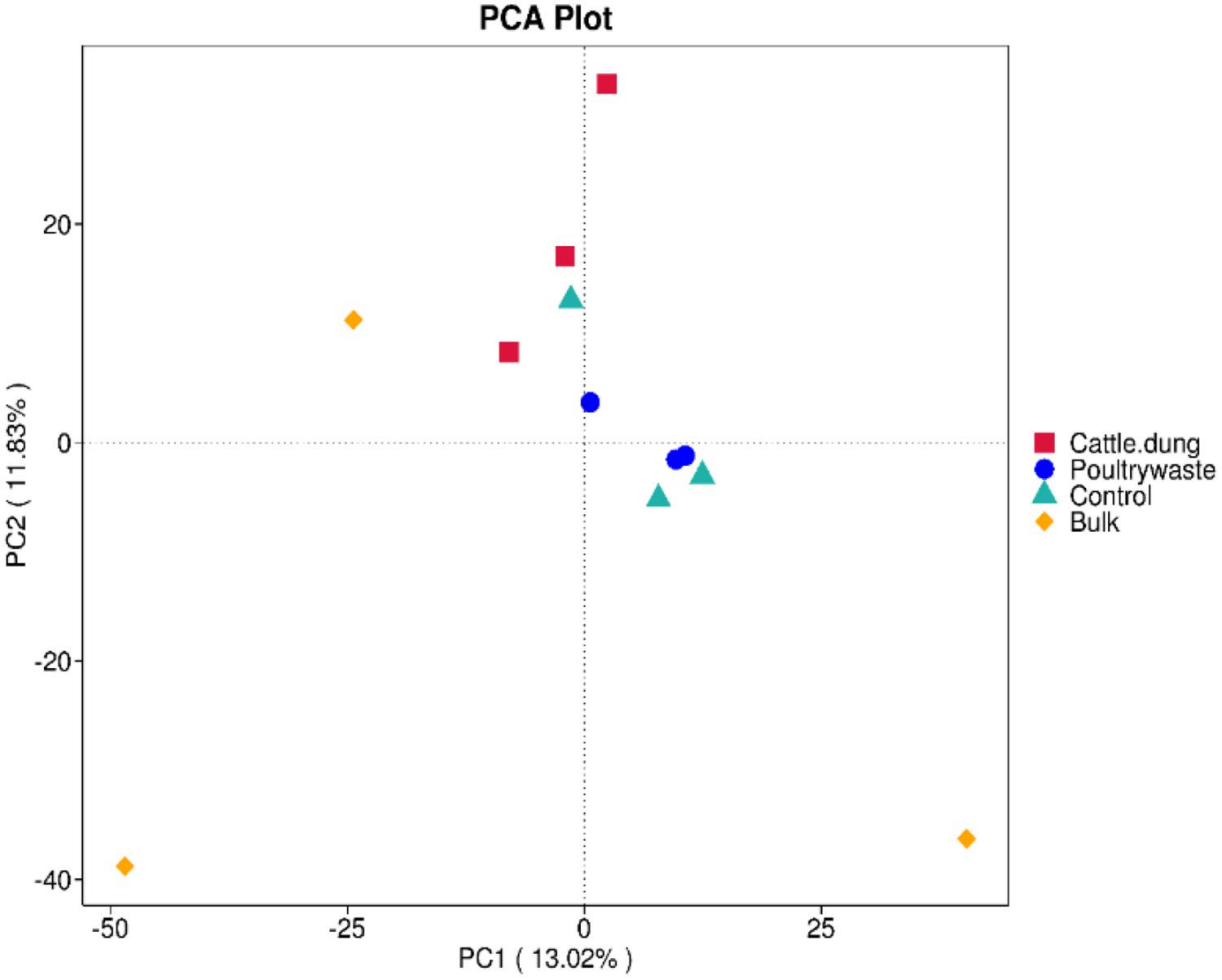
Principal component analysis (PCA) plot of fungal community distribution across the treatments.

Principal coordinate analysis (PCoA) based on the Weighted UniFrac and Unweighted Unifrac distances was performed to assess treatment‐driven differences in community structure (Figure 8). The Weighted UniFrac plot (Figure 8a) accounted for the presence and relative abundance of taxa, where both PC1 (29.12%) and PC2 (23.49%) accounted for significant variation in the fungal communities. The cattle dung samples clustered distinctly along the positive side of PC2, poultry waste forming more distinct groupings, indicating that organic inputs strongly shape fungal community composition, while control and bulk soil samples also separated in PC2. In contrast, the Unweighted Unifrac plot (Figure 8b), with PC1 (21.5%) and PC2 (12.62%), shows a lower variance contribution. Cattle dung, poultry waste, bulk, and control samples appeared more dispersed, with partial overlaps but also distinct shifts along both axes. This pattern indicates that while cattle dung strongly influenced abundant taxa (Weighted UniFrac), all treatments altered the presence of rare taxa, leading to broader community‐level differences (Unweighted UniFrac). The UPGMA cluster tree calculated with the Weighted Unifrac distance (Figure S1) indicates that all treatments had different fungal communities, and poultry waste‐amended soil forms a distinct cluster; a visible cluster of samples in poultry waste and cattle dung treatments implies more comparable community arrangements within each amendment type, while control and bulk soils show greater variability (Figure S2).

**Figure 8:**
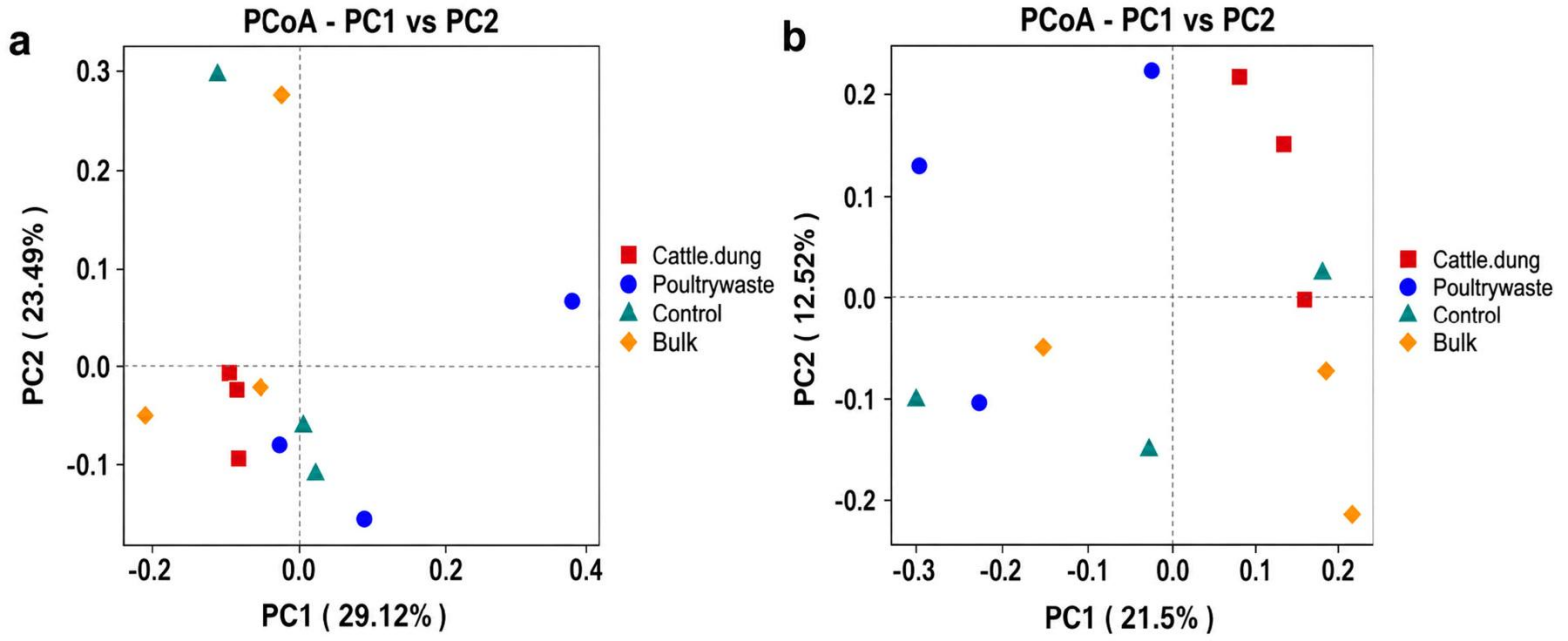
PCoA based on (a) weighted unifrac distance and (b) unweighted unifrac distance.

To check for differences in soil fungi caused by the different soil treatments, ANOSIM (Table 2) was run on the soil samples from cattle dung, poultry waste, control, and bulk soil treatments. Results are measured by R‐values that indicate how distinct the groups are (from −1 to 1) and P‐values that indicate whether the results are statistically significant. Of all the comparisons, the highest R‐value was found for cattle dung versus poultry waste (*R* = 0.74). Moderate differences were also seen between cattle dung and bulk (*R* = 0.41, *p* = 0.1) and between poultry waste and bulk (*R* = 0.33, *p* = 0.1). By contrast, poultry waste versus control (*R* = −0.07, *p* = 0.8) and control versus bulk (*R* = −0.44, *P* = 1) showed little to no separation in fungal composition. Overall, some pairwise comparisons suggest amendment‐associated shifts in the microbiome, particularly for cattle dung and poultry waste, but none reached conventional statistical significance (*p* < 0.05).

**Table 2:** ANOSIM results of microbial community differences among treatments.

Group	R‐value	*p* value
Cattle dung – Poultry waste	0.74074	0.10
Cattle dung – Control	0.33333	0.30
Cattle dung – Bulk	0.40741	0.10
Poultry waste – Control	−0.07407	0.80
Poultry waste – Bulk	0.33333	0.10
Control – Bulk	−0.44444	1.00

The corresponding multi‐response permutation procedure (MRPP) analysis (Table S3) corroborated the ANOSIM results: the largest dissimilarity again occurred between cattle dung and poultry waste, and none of the pairwise comparisons reached conventional statistical significance (*p* = 0.1–0.8).

Figure 9 presents the LEfSe cladogram, mapping the phylogenetic placement and statistical support (LDA scores) of the fungal lineages that most strongly differentiated the four treatments. The number of fungi in the group Hypocreales was highest in cattle dung‐treated soils. Soil fertility and plant‐supportive traits are often linked to Hypocreales, which may therefore contribute to improving soil health under cattle dung fertilization. Alternatively, the Onygenales group appeared more often in samples of poultry waste‐treated soils, suggesting a preference for poultry amendments in their growth; many Onygenales decompose keratin waste, which coincides with the composition of poultry waste. For most other fungal clades, there was no significant difference in their presence between the groups, indicating a similar fungal core across all samples.

**Figure 9:**
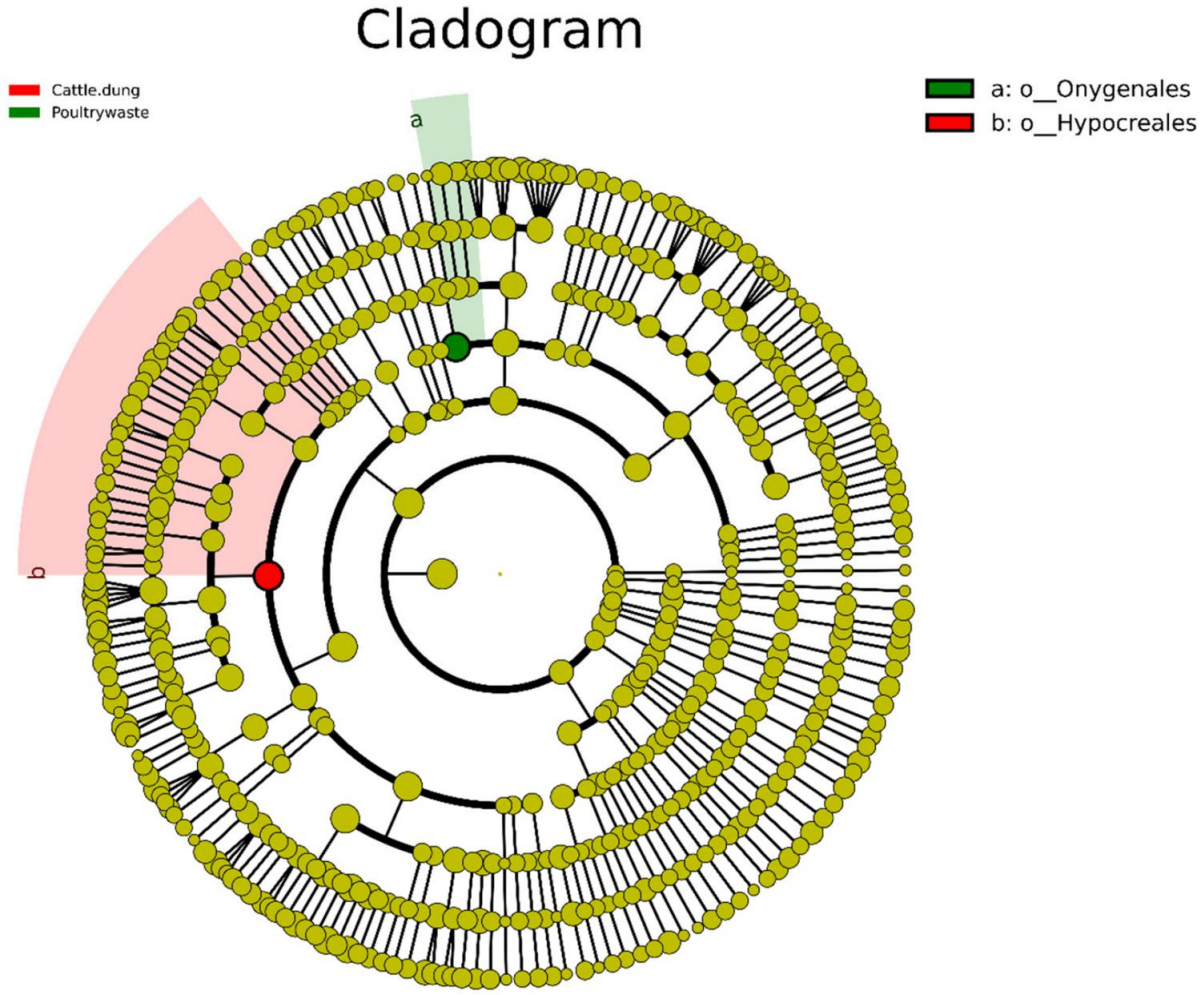
Cladogram of fungal abundance among treatments (Cattle dung, Poultry waste, Control, Bulk).

The FunGuild heatmap presents a comparative overview of fungal functional guilds across the four treatments (Figure 10a). Cattle dung exhibited strong enrichment for saprotrophic and pathogenic guilds, particularly Dung_Saprotrophs, Animal_Pathogens, and Wood_Saprotrophs. Poultry waste showed a distinct enrichment of Endophyte and Plant_Pathogen guilds. Untreated control demonstrated a lower level of saprotrophs and pathogens compared to amended treatments, while bulk soil samples clustered separately, showing enrichment for Wood_Saprotrophs and Ectomycorrhizal fungi. The corresponding Venn diagram of FunGuild classifications (Figure 10b) identified 32 core functional guilds common to all treatments, representing a stable set of ecological functions important for soil health and soybean growth, with minimal treatment‐specific unique guilds (3 in bulk soil, 1 in control, none in cattle dung or poultry waste). Together, these results indicate that organic fertilization has a genus‐ and guild‐specific effect on the distribution of fungal function in the soybean rhizosphere, superimposed on a conserved functional core.

**Figure 10:**
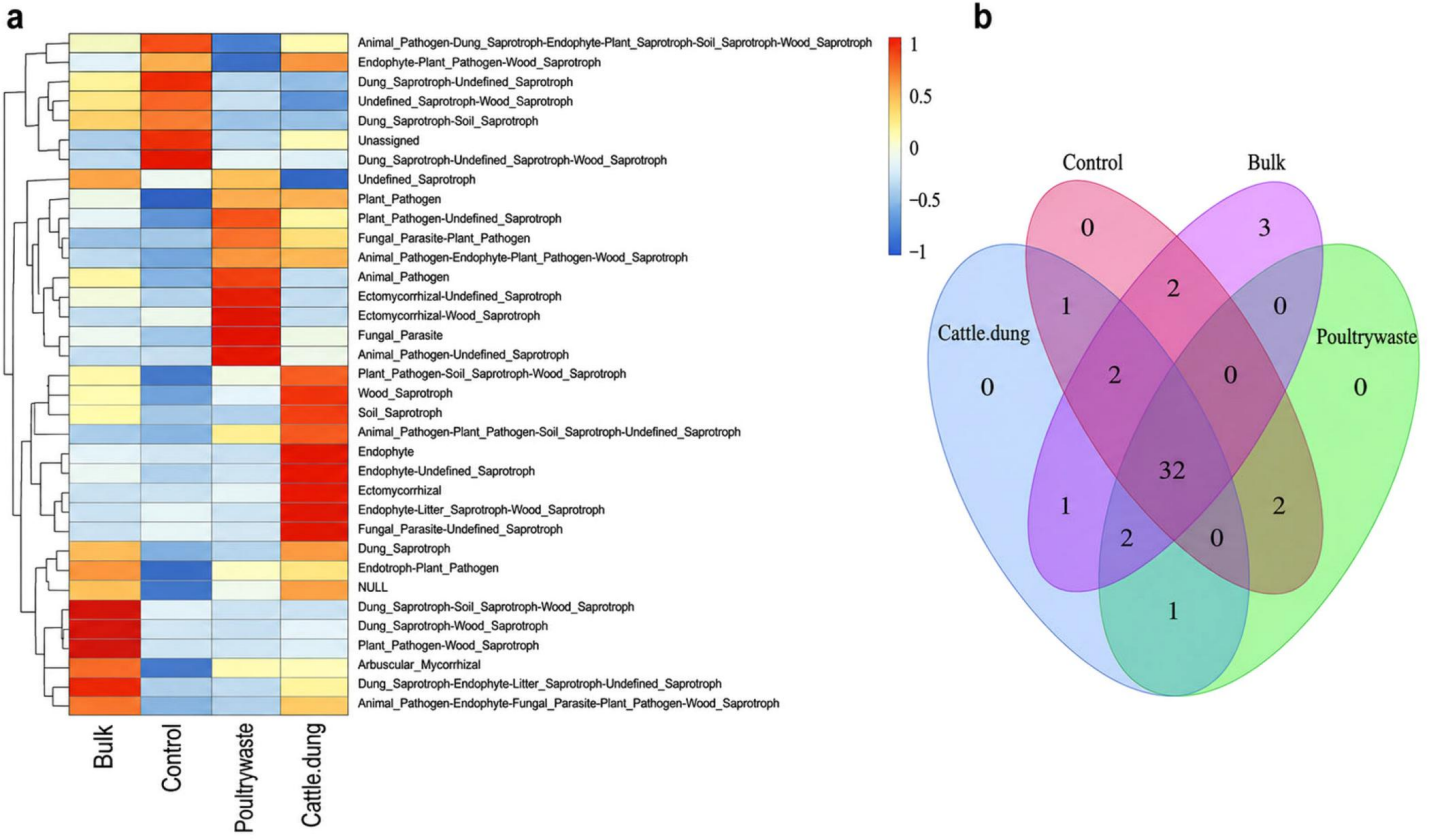
(a) Functional guild distribution of fungal communities across treatments and (b) venn diagram of FunGuild results.

Figure S4 shows a T‐test based gradient analysis of cattle dung and poultry waste. Actinobacteria, Firmicutes, and Proteobacteria grew better in poultry waste‐treated soils, whereas control samples contained fewer taxa associated with nutrient breakdown, indicating that organic amendment supports the growth of such microbial groups. Sample‐ and taxon‐based clustering identified shared microbial communities between bulk and poultry waste treatments, while each treatment also retained its own distinct taxonomic clusters. Figure S4b shows Curvularia and Parazalerion abundance in the control and bulk treatment groups: Curvularia was significantly more abundant in bulk soil (*p* = 0.001), while Parazalerion, though rare overall, showed a marginally significant decrease in bulk soil (*p* = 0.032), with limited ecological impact given its low overall abundance.

The SIMPER analysis of the 10 most influential microbial pathways across the 12 soil samples identified carbohydrate metabolism and amino acid metabolism as the largest contributors to community dissimilarity (contribution 0.15–0.17), reflecting the central role of carbohydrates and proteins in microbial nutrient transformation and energy production. Nucleotide metabolism, cofactor and vitamin metabolism, and energy metabolism contributed moderately across most samples, consistent with general metabolic adaptability, whereas lipid metabolism, glycan biosynthesis, and cell motility contributed comparatively little (below 0.05), suggesting a more restricted or context‐dependent role (Figure S5).

## 4 Discussion

This study is an integrative investigation on the compositionality, diversity, and functional guild distribution of fungal communities on soybean rhizosphere modified with cattle dung and poultry waste treatment, unamended control, and bulk soils. It has been reported that organic fertilization has a strong treatment‐specific influence on fungal assemblages, determining the dominant taxa and functional guilds as indicated by ITS‐based amplicon sequencing and several ecological analyses (Iqbal et al. 2022; Gao et al. 2022). Thus, this observation throws some light on organic inputs modulating rhizosphere microbial ecology with consequences for sustainable soil fertility management in soybean production systems (Wijayawardene et al. 2024; Kayiranga et al. 2024).

Cattle dung and poultry waste manure supported different fungal lineages at the phylum level. Cattle manure enhanced the relative abundance of phyla within Chytridiomycota, Aphelidiomycota, and Rozellomycota; these phyla are often saprotrophic and cellulose‐degrading (Wijayawardene et al. 2024). This gives further evidence that moisture is enabling the litter to select taxa adapted to degradation of recalcitrant organic matter (Gao et al. 2022; Huang et al. (2012). Nutrient‐dense poultry manure spurred copiotrophs and fast‐growing saprobes, with Mortierellomycota and Ascomycota dominating poultry residues (Iqbal et al. 2022; Wu et al. 2025). Mortierellomycetes have been noted as active nitrogen mineralizers and phosphate solubilizers, thus implying that nutrient cycling potential under poultry manure development would differ from that of cattle dung (Zhu et al. 2024; Silva et al. 2023). In marked contrast, the control soils and bulk were characterized by high abundances of Olpidiomycota, Zoopagomycota, and Kickxellomycota, taxonomically associated with soils that either took up little nitrogen or were minimally disturbed (Wijayawardene et al. 2024; Zhang et al. 2019). The stress tolerance and reduced function of this community have been interpreted in the context of the relatively low nutrient status of unamended soils (Gao et al. 2022; Kayiranga et al. 2024).

Such patterns of colony distribution reveal the ecological pressures imposed by organic amendments upon the genus. Cattle dung stimulates such filamentous Ascomycete genera as Chaetomium, Neurospora, and Humicola, which are thought to play an important role in cellulose degradation and organic matter turnover in soils (Zhang et al. 2022; Huang et al. 2012). Trichoderma and Penicillium are the two genera known to promote plant growth, provide biocontrol mechanisms, and enhance nutrient flow to plants, which were favoured by poultry wastes (Cui et al. 2023; Iqbal et al. 2022). Control and bulk soils displayed predominance of Fusarium, Curvularia, and Stagonosporopsis. In addition, the fact that nutrient‐poor soils were often dominated by these organisms further denotes the suppressive role organic amendments typically exert against potentially harmful taxa, a fact consistent with ecological theories in which organic inputs shift the focus of competitive hierarchies toward beneficial saprotrophs (Gao et al. 2022; Kayiranga et al. 2024).

The Venn analysis corroborated these community distinctions, showing that the treatments have unique, though not wholly exclusive, ASVs. The highest unique ASV counts were from bulk soil and cattle dung‐amended soils. Altogether, the presence of a common core of 300 ASVs indicates the persistence of fungal taxa independent of treatment in the soybean rhizosphere, likely reflecting host root exudates and plant‐mediated filtering processes (Kayiranga et al. 2024; Wang and Zou 2024). Alpha diversity indices provided the clearest and statistically most robust evidence of treatment effects on species richness and evenness among all the community‐level metrics examined in this study. Bulk soil had the highest richness, likely because it is largely undisturbed, whereas poultry amendment expressed the lowest richness and evenness, possibly reflecting nutrient‐driven ecological filtering that favours fast‐growing copiotrophs over less abundant taxa (Tang et al. 2023). In contrast, cattle dung‐treated soils had the most favourable Shannon and Simpson values among the amended soils, pointing toward a relatively balanced and heterogeneous community likely driven by the diverse organic substrates present in cattle manure (Gao et al. 2022; Zhang et al. 2022). These patterns further demonstrate that the type of organic input, not merely the presence of organic material, is important in shaping the diversity of the rhizosphere fungi (Wijayawardene et al. 2024).

Beta‐diversity and ordination clearly indicated compositional shifts brought about by the amendments commonly used in organic farming. Strong dissimilarity of poultry waste from both cattle dung and control soil indicates a substantial restructuring effect on the fungal communities under these conditions (Iqbal et al. 2022; Cui et al. 2023). PCA and PCoA results showed clear clustering of communities from cattle dung and poultry waste treatments, while bulk and control soils showed partial overlap and greater heterogeneity. For the weighted metric, cattle dung had one of the strongest effects on abundant taxa, whereas the unweighted metric revealed a greater shift in rare taxa across all treatments, pointing to possible long‐term consequences for soil‐level resilience and functional stability (Yang et al. 2024).

ANOSIM and MRPP results indicated no significant treatment differences at conventional threshold values; however, the moderate R‐values and effect sizes, most notably between cattle dung and poultry waste treatments, suggest ecologically meaningful but statistically subtle divergences. This is not surprising, as soil variability, the limited number of field replicates (n = 3; see Statistical analysis), and complex interactions between manure type, indigenous microbiota, and plant roots may obscure statistically significant separation (Yang et al. 2022; Gao et al. 2022). It is important to distinguish these ecologically suggestive but statistically non‐significant patterns from the formally significant alpha‐diversity findings reported above: the beta‐diversity, ANOSIM, and MRPP results should be interpreted as trends warranting further investigation rather than as confirmed treatment effects, whereas the alpha‐diversity comparisons, which showed clear and consistent separation among treatments, represent the most statistically robust conclusions of this study.

Functional predictions via FUNGuild offered further insight into the ecological implications of organic fertilization. Cattle dung was strongly associated with dung saprotrophs and wood decomposers, consistent with the taxonomic dominance of cellulose‐degrading Ascomycetes (Nguyen et al. 2016; Binder et al. 2016). Poultry manure showed a distinct enrichment of plant pathogens and endophytes, likely reflecting the nitrogen‐rich and keratin‐rich character of poultry waste and its association with Onygenales and other keratinolytic fungi (Kandemir et al. 2022; Petek et al. 2024). Bulk soils were enriched for ectomycorrhizal and wood‐decaying guilds, indicating a natural soil baseline with broader ecological breadth. Nonetheless, all treatments shared 32 functional guilds, further supporting the presence of a stable core set of ecological functions in the soybean rhizosphere (Nguyen et al. 2016). Given the marginal statistical support from ANOSIM and MRPP for community‐level differences, these FUNGuild‐based functional distinctions, together with the LEfSe findings discussed below, are best regarded as exploratory and hypothesis‐generating rather than as definitive evidence of treatment effects, and warrant confirmation with greater replication.

LEfSe outcomes also supported the observed taxonomic and functional patterns, with Hypocreales as a marker for cattle dung and Onygenales for poultry waste. Hypocreales are generally among the most represented beneficial fungal groups in soil and include taxa used for biocontrol, suggesting that cattle dung amendment may support soil health and disease suppression (Gao et al. 2022; Zhang et al. 2022). The keratin‐degrading Onygenales are notably diverse in poultry‐rich soils, complementing the biochemical characteristics of poultry manure and indicating a distinct decomposition pathway (Petek et al. 2024; Labuda et al. 2024). Beyond identifying these two biomarker taxa, the cladogram (Figure 9) situates them within their broader phylogenetic context, allowing the treatment‐associated enrichment to be interpreted alongside neighbouring lineages, which strengthens confidence that the signal reflects coherent clades rather than isolated taxa; as with the FUNGuild patterns above, however, these LEfSe‐based associations should be treated as hypothesis‐generating rather than confirmatory. Finally, predicted metabolic pathway outcomes confirmed that carbohydrate and amino acid metabolism predominate across treatments, corroborating that rhizosphere function fundamentally relies on carbon and nitrogen turnover (Iqbal et al. 2022, da Silva et al. 2023). The relatively lower contribution of lipid metabolism and glycan biosynthesis suggests that these pathways are of secondary importance under organic fertilization.

It is also important to acknowledge the potential confounding role of underlying soil physicochemical variability. The physicochemical analysis (Table S1) showed that the four treatments differed appreciably in extractable P, Ca, K, Mg, Na, and pH, independent of the applied organic amendment. Because these properties were not statistically controlled for in the diversity or ordination analyses presented here, they may partly confound the interpretation of amendment effects on the fungal community. Future studies should consider incorporating these soil properties as covariates, or stratifying analyses by soil physicochemical clusters, to more clearly separate the direct effects of organic amendment from indirect effects mediated by pre‐existing or amendment‐induced soil chemistry.

Conclusively, these results indicate, most robustly at the level of alpha diversity, that organic fertilizers are associated with changes in the community structure, biodiversity, and ecological functioning of fungi in the rhizosphere of soybean. Cattle dung tends to encourage the establishment of diverse and functionally rich saprotrophic communities, which are probably beneficial to soil health, while poultry manure typifies more specialized communities, dominated by nutrient‐responsive taxa. The community‐composition and FUNGuild‐based functional differences, however, should be regarded as exploratory and hypothesis‐generating, given their marginal statistical support from ANOSIM and MRPP, pending confirmation with greater replication. Thus, different types of fertilizer appear to act differently on microbial ecological outcomes.

## Author Contributions

**Ijeoma Emelda Osuji:** methodology, software, investigation, visualization, formal analysis, writing – original draft, writing – review and editing. **Akinlolu Olalekan Akanmu:** methodology, investigation, software, formal analysis, co‐supervision, visualization, data curation, writing – review and editing. **Olubukola Oluranti Babalola:** conceptualization, supervision, software, investigation, visualization, writing – review and editing, funding acquisition, formal analysis, resources.

## Ethics Statement

The current work follows the ethical requirements for publication. It does not involve human subjects, animal experiments, or any data collected from social media platforms.

## Conflicts of Interest

The authors declare no conflicts of interest.

## Supporting information


**Figure S1:** UPGMA cluster tree based on Weighted Unifrac distance.
**Figure S2:** NMDS based on Weighted Unifrac distance (a) and Unweighted Unifrac distance (b).
**Figure S3a:** MetagenomeSeq analysis between groups (Cattle dung ‐ Poultry waste).
**Figure S3b:** MetagenomeSeq analysis between groups (Control ‐ Bulk).
**Figure S4:** T‐test (Cattle dung, Poultry waste).
**Figure 4b:** T‐test of control and bulk.
**Figure S5:** Simper pathways of microbial samples.
**Table S1:** Physical and chemical properties of the rhizosphere and bulk soil samples.
**Table S2:** Sequencing data processing statistics of soybean treatments.
**Table S3:** Multi‐Response Permutation Procedure (MRPP) analysis result.

## Data Availability

The raw sequences have been deposited with NCBI under the BioProject accession number PRJNA1177223 (https://www.ncbi.nlm.nih.gov/bioproject/PRJNA1177223).
