# Supplementary material for: Amplicon Characterization of Fungal Diversity and Functional Prediction of Glycine max L. Rhizosphere Microbiome Under Different Organic Fertilization
Source: Microbiologyopen. 2026 Oct 8;15(5):e70437. doi: 10.1002/mbo3.70437 (PMC13650260; doi:10.1002/mbo3.70437)
Supplement: Supplementary file 1 [file MBO3-15-e70437-s001.docx]

Supplementary Table 1. The physical and chemical properties of the rhizosphere and bulk soil samples.

| **Analyte** | **Cattle dung** | **Poultry waste** | **Bulk** | **Control** |
| --- | --- | --- | --- | --- |
| Clay (%) | 18.11▒±▒0.18b | 18.05▒±▒0.32c | 16.17▒±▒0.15▒d | 18.16▒±▒0.12a |
| Sand (%) | 77.98▒±▒0.07a | 77.98▒±▒0.08a | 78.06▒±▒0.04a | 77.97▒±▒0.11a |
| Silt (%) | 4.01▒±▒0.09b | 4.01▒±▒0.03b | 6.00▒±▒0.10a | 3.90▒±▒0.12b |
| P (mg/kg) | 27.88▒±▒0.28b | 34.17▒±▒0.31a | 10.20▒±▒0.23▒d | 15.12▒±▒0.32c |
| Na (mg/kg) | 28.11▒±▒0.14b | 27.98▒±▒0.07b | 25.13▒±▒0.12c | 44.03▒±▒0.31a |
| K (mg/kg) | 21.00▒±▒0.11a | 13.00▒±▒0.11c | 12.92▒±▒0.16▒d | 17.98▒±▒1.03b |
| Ca (mg/kg) | 46.95▒±▒0.20b | 48.01▒±▒0.04a | 32.94▒±▒0.06▒d | 41.94▒±▒0.14c |
| Mg (mg/kg) | 23.99▒±▒0.06a | 14.01▒±▒0.08▒d | 16.64▒±▒0.40c | 22.62▒±▒0.42b |
| pH | 7.29▒±▒0.02a | 7.25▒±▒0.24b | 7.27▒±▒0.32b | 7.79▒±▒0.04a |
| Na (cmol/kg) | 0.12▒±▒0.00b | 0.12▒±▒0.00b | 0.11▒±▒0.00c | 0.19▒±▒0.00a |
| K (cmol/kg) | 0.53▒±▒0.00▒d | 0.34▒±▒0.00b | 0.32▒±▒0.00a | 0.43▒±▒0.00c |
| Ca (cmol/kg) | 2.32▒±▒0.00b | 2.40▒±▒0.01a | 1.61▒±▒0.01▒d | 2.04▒±▒0.00c |
| Mg (cmol/kg) | 1.91▒±▒0.08a | 1.17▒±▒0.02c | 1.82▒±▒0.05b | 1.82▒±▒0.00b |
| EC (mS/m) | 48.41▒±▒0.02▒d | 48.98▒±▒0.10c | 58.95▒±▒0.10a | 49.01▒±▒0.19b |
| NH4▒+▒-N (mS/m) | 8.98▒±▒0.19a | 6.09▒±▒0.20b | 6.72▒±▒0.04c | 5.18▒±▒0.14▒d |
| NO3-N (mg/kg) | 11.08▒±▒0.29c | 13.25▒±▒0.05a | 5.84▒±▒0.18▒d | 12.09▒±▒0.11b |

Means with different letters across the row are significantly (p▒<▒0.05) different from one another.

Supplementary Table 2. Sequencing data processing of soybean treatment.

| **Samples** | **RawPE** | **Combined** | **Qualified** | **Nochime** | **Base (nt)** | **Avglen (nt)** | **GC (%)** | **Q20 (%)** | **Q30 (%)** |
| --- | --- | --- | --- | --- | --- | --- | --- | --- | --- |
| Cattle dung | 185049.5 | 181846.7 | 181506.8 | 164407.3 | 39529816 | 242.60 | 51.65 | 99.51 | 97.86 |
| Poultry waste | 191552.3 | 188094.5 | 187772.3 | 171701 | 41190752 | 254.56 | 53.66 | 99.37 | 97.33 |
| Untreated control | 188300.9 | 184970.6 | 184639.5 | 168054.2 | 40360284 | 240.88 | 51.40 | 99.48 | 97.73 |
| Bulk | 188300.9 | 184970.6 | 184639.5 | 168054.2 | 40360284 | 239.47 | 51.05 | 99.51 | 97.84 |

Supplementary Table 3. Multi-Response Permutation Procedure (MRPP) analysis result.

| **Group** | **A** | **observed-delta** | **expected-delta** | **Significance** |
| --- | --- | --- | --- | --- |
| Cattle dung – Poultry waste | 0.10051 | 0.61153 | 0.67987 | 0.1 |
| Cattle dung – Control | 0.04732 | 0.64019 | 0.67199 | 0.1 |
| Cattle dung – Bulk | 0.04871 | 0.57961 | 0.60929 | 0.1 |
| Poultry waste – Control | 0.00287 | 0.6928 | 0.6948 | 0.5 |
| Poultry waste – Bulk | 0.03961 | 0.63222 | 0.6583 | 0.2 |
| Control – Bulk | -0.04061 | 0.66088 | 0.63509 | 0.8 |

Figurementary Figure 1: UPGMA cluster tree based on Weighted Unifrac distance

Figurementary Figure 2: NMDS based on Weighted Unifrac distance (a) and Unweighted Unifrac distance (b)

Figurementary Figure 3a: MetagenomeSeq analysis between groups (Cattle dung - Poultry waste)

Figurementary Figure 3b: MetagenomeSeq analysis between groups (Control - Bulk)

Figurementary Figure 4: T-test (Cattle dung, Poultry waste)

Figurementary Figure 4b: T-test of control and bulk

Figurementary Figure 5: Simper pathways of microbial samples
